# The role of extra-familial factors in adolescence for the association between out-of-home care and adult psychiatric disorders–A birth cohort study

**DOI:** 10.1371/journal.pone.0318231

**Published:** 2025-01-28

**Authors:** Lisa Bornscheuer, Evelina Landstedt, Ylva B. Almquist

**Affiliations:** 1 Department of Public Health Sciences, Stockholm University, Stockholm, Sweden; 2 Department of Social and Psychological Studies, Karlstad University, Karlstad, Sweden; Public Library of Science, UNITED KINGDOM OF GREAT BRITAIN AND NORTHERN IRELAND

## Abstract

**Background:**

Psychiatric disorders are a substantial public health concern, and childhood adversity a well-known risk factor for it. Investigating gender differences in vulnerability and resilience processes following out-of-home care (OHC) as proxy for childhood adversity can help map opportunities for the prevention of psychiatric disorders.

**Methods:**

We followed a large birth cohort for psychiatric disorders (anxiety, depression, and self-harm, and substance misuse) between age 25–62 years, comparing individuals with and without OHC experience. We investigated different extra-familial risk and resources factors following OHC via gender-stratified mediation and moderation analyses to approximate risk accumulation (vulnerability processes) and buffers of risk (resilience processes).

**Results:**

OHC is prospectively associated with psychiatric disorders in adulthood. Lower school grades, delinquency, and early parenthood are mediators of the association, with the exception of education in girls in relation to anxiety, depression, and self-harm, and early parenthood in boys in relation to substance misuse. Number of best friends moderates OHC experience in boys, and there is a trend for higher educational achievement to also act as buffer, even though this trend was not statistically significant. Leisure time activities did not act as buffer.

**Conclusion:**

Vulnerability and resilience processes after childhood adversity are gendered: Risk accumulation runs via delinquency and poorer educational outcomes in boys more than in girls, while early parenthood is a more dominant risk factor in girls. Having more best friends and higher grades may act as buffer, especially in boys.

## Introduction

Mental ill-health causes a substantial burden, both on an individual level and in terms of costs to society and healthcare systems [1, 2]. One step towards reducing the burden is to understand how to break the vulnerability processes leading up to mental ill-health, as well as understanding how to foster resilience processes. The former goal can be achieved by preventing accumulation of risk factors, while the latter goal translates into increasing availability of resources that promote mental health, especially for those at increased risk. If a resource is particularly beneficial for individuals at higher risk, it is a buffer of that risk. Against this backdrop, this study focuses on severe childhood adversity, indicated by out-of-home care (OHC) [3], as predictor of psychiatric disorders across the life course [4], and extra-familial risk and resource factors that might matter for this association, with a particular interest in gender patterns.

Childhood is a period in which violations of the expectable environment, such as dysfunction in the family, can come to shape physiological and behavioural stress responses in ways that are maladaptive [5–7]. A life course framework poses that early adversity can thus accumulate with time and/or trigger chains of risk, which can lead to lasting disadvantage [8]. Previous empirical research on the accumulation of risk following early adversity has identified various pathways to mental ill-health, with poor educational outcomes emerging as one significant factor [4, 9]. Early adversity is related to an increased likelihood of lower educational achievement, which in turn often translates into poorer labour market outcomes [10]. Considering the socioeconomic gradient present in nearly all health outcomes, and the role that the distribution of social and material resources plays in contributing to health inequalities, it is plausible that education serves as a significant mediator between early adversity and later mental ill-health [11]. Relatedly, other extra-familial factors that can interfere with educational achievement, such as early parenthood and delinquency, are relevant to analyses of vulnerability processes, especially from a gender perspective. For example, early parenthood is more common in individuals with experience of childhood adversity [12], and can prevent especially young mothers from establishing themselves in the labour market [13, 14]. Furthermore, motherhood in adolescence increases the risk of poor health outcomes, especially in conjunction with low social support [15–17]. Similarly, delinquency and poor mental health are associated [18, 19], but delinquency is a risk factor that is more frequently observed in boys than girls [20]. It is also more prevalent among those with experience of childhood adversity [21]. Both early parenthood and delinquency therefore constitute promising candidates for examining gendered risk accumulation following adversity.

While early adversity is a strong risk factor, it is not deterministic [8, 22]. Resilience research therefore explores heterogeneity in outcomes after severe stress exposure, and investigates amongst other things potential protective factors that can even out said stress exposures [23]. Resilience refers to multi-domain processes that include individual-level resources, but also relationships and the wider ecological systems theses are embedded in [23, 24]. Since OHC implies disconnection from the family, and thereby restricted access to resources in the home, it is particularly relevant to explore extra-familial resources as buffers of adversity. Previous research indicates that social support by non-family members is such a buffer [25], and possibly more so among women than among men [25, 26]. Leisure time activities have also proven beneficial for young people from disadvantaged backgrounds [27]. Both close friendships and meaningful leisure time activities can provide youth with a sense of connection and belonging that are said to promote resilience [28–30]. Lastly, better educational outcomes—as the flip-side of the risk imposed by poor educational outcomes—represents another potential buffer. On the one hand, education is an overarching resource and a strong predictor of future socioeconomic status [11]. On the other hand, education also provides more specific resources, such as health literacy [31].

There is a considerable amount of research on the pathways along which risks accumulate after exposure to childhood adversity, as well as on factors that buffer the impact of such adversity. In studies of mental ill-health, the former has primarily been investigated by means of mediation analysis, whereas the latter typically is addressed through moderation analyses. Despite well-documented gender patterns of both mental ill-health and the distribution of risk and resource factors [24, 32], there is a lack of research on how gender might influence the interplay between such factors and mental health outcomes following childhood adversity [24, 33]. This is a research gap this study sets out to fill. Furthermore, even though empirical evidence concerning many potential mediators and moderators of adversity exists, this evidence is often inconsistent across studies [33, 34]. Additionally, existing studies are highly heterogeneous. Consequently, there is a general interest in strengthening–or refuting–current evidence on mediators and moderators of childhood adversity in different contexts [34], identifying the pathways along which vulnerability unfolds, as well as the resource factors which can foster resilience. Lastly, there is a need for prospective studies based on community-based samples to complement existing research, which is often limited to retrospective data and non-representative samples. In the current study, we follow a large cohort of individuals from birth up until retirement, leveraging both register and survey data.

1. The aim of the study is to better understand the accumulation of risk (approached as mediation) and the overcoming of risk (approached as moderation) incurred by childhood adversity, with particular attention to gender patterns. We address the following research questions:
2. Do extra-familial risk factors in adolescence (delinquency, early parenthood, grades) mediate the association between OHC experience and psychiatric disorders in adulthood?
3. Do extra-familial resource factors in adolescence (number of best friends, leisure time activities, grades) moderate the association between OHC experience and psychiatric disorders in adulthood?
4. Are there gender patterns in regard to 1) or 2)?

## Methods

### Data

Data stem from the Stockholm Birth Cohort Multigenerational Study (SBC Multigen), a cohort of individuals born in 1953, and living in the Stockholm metropolitan area in 1963. The current study relied on data from several national registers, and from a school survey conducted in 1966. Inclusion criteria were: individuals had to be alive at the beginning of follow-up (1978–2015), registered in Sweden for at least one year during follow-up, have complete information on all key variables, had to be in year 6 during the time of the school survey, and not have had the first placement in OHC after age 12. This resulted in a sample size of n = 11,011 (see S1 Fig and S1 Table in S1 File for further information). All main analyses were conducted as complete case analyses.

Data were obtained on 08 February 2018; for more detailed information on the data material please refer to Almquist et al. [35]. Work on the study presented here began on 08 October 2023.

### Ethics

The creation of the dataset used in this study was approved by the Regional Ethical Review Board in Stockholm (no. 2017/34-31/5; 2017/684-32). The study itself is covered by an additional ethical approval (no. 2019–04376), granted by the same body. The datasets generated and/or analysed during the current study are not publicly available due to lacking ethical approval for data sharing, as restricted by the Swedish Ethical Review Authority (registrator@etikprovning.se). Interested researchers are encouraged to directly apply to the registry holders in Sweden. Data are pseudonymized, meaning that the authors are not able to identify individual participants. Consent was waived for this study, as is often the case for register-based studies, since participants remain unidentifiable throughout all stages of research and research communication, and the benefits of research conducted in the public interest using rich routine data material is deemed to outweigh possible risks by far.

### Variables

#### Outcome variables

Psychiatric disorders (age 25–62 years) were operationalised as two outcomes: a) inpatient care related to diagnoses of anxiety, depression and self-harm (ADS), and b) inpatient care related to substance misuse (SM), including alcohol-related chronic diseases. We used data from inpatient care recorded in the National Patient Register, and the outcomes were coded 0/1 (no/at least one diagnosis). The International Classification of Disease (ICD-10) codes for included diagnoses are listed in the supporting information (S2 Table in S1 File). Inpatient care covers cases where the patient has been hospitalised, but does not include cases where, for example, there has been a contact with acute care that did not result in a hospitalisation.

#### Exposure

OHC (age 0–12 years) was operationalised as placement into residential care or family foster care due to reasons related to the biological parents. Data were retrieved from the Social Registers.

#### Mediators, moderators, and gender

Extra-familial risk factors (mediators): Delinquency (ages 14–21), early parenthood (ages 14–21), and grades (age 13). Data was retrieved from the National Crime Register, the Total Population register, and the Marks register.

Extra-familial resource factors (moderators): Number of best friends (age 13), leisure time activities (age 13), and grades (age 13). Data were retrieved from the School Survey and the Marks register.

Gender: We used sex registered in the Total Population Register (male/female) as proxy for gender.

More detailed information on extra-familial risk and resource factors can be found in the supporting information (S3 Table in S1 File).

### Statistical analyses

Descriptive analyses include frequencies and percentages of the study variables by gender as well as the prevalence of mental ill-health (ADS and SM, respectively) across these variables. Logistic regressions were used to estimate bivariate associations between the mediators and psychiatric disorders as well as between the moderators and mental ill-health.

Mediation analyses were performed by means of logistic regression analysis based on the Karlson-Holm-Breen (KHB) method. We report the significance of the total, direct and indirect effects, as well as the mediation percentage (MP). Cross-model comparisons of non-linear models are prone to bias, and the KHB method remains unaffected by this problem [36]. It is a form of decomposition analysis suitable for splitting a total effect into a direct and an indirect effect via included mediators [36].

Interaction analyses were employed to assess moderation, all based on logistic regression modelling. We investigated both two-way interaction terms (OHC x moderator) and three-way interaction terms (OHC x moderator x gender). From the interaction analyses, we present p-values from likelihood-ratio tests (LRT) comparing the models with and without the interaction terms, to assess whether the model with interaction terms fits the data better. We also present margin plots of the interactions, stratified by gender.

All analyses, except for the three-way interaction analysis, were conducted in the overall sample as well as stratified by gender.

#### Sensitivity analyses

The KHB method does not extend to event history analysis (such as Cox regression modelling), meaning that we could not account for time under risk. We therefore provide results from bivariate Cox regression models in the supporting information (S4a and S4b Tables in S1 File), which show that the magnitude and statistical significance of the estimated associations do not differ substantially between Cox and logistic regression analysis.

We considered parental socioeconomic status (SES) as a possible confounder. Although most childhood conditions are difficult to disentangle from OHC placement, also children with OHC experience were in their home environments for some time. It is known that parental SES is associated with increased likelihood of various adversities [37], as well as with poor mental health outcomes later in life [38]. We therefore re-ran all bivariate analyses between mediators, moderators, and both outcomes adjusting for parental SES (results not reported). Parental SES was not associated with ADS, but in some analyses increasing parental SES reduced the likelihood of SM. However, including parental SES did not substantially change the magnitude of the associations between mediators/moderators and psychiatric disorders.

## Results

Table 1 shows that a significantly greater proportion of men and women with OHC exposure were diagnosed with a psychiatric disorder compared to those without OHC exposure. In the total sample and in boys, lower school grades, delinquency, and early parenthood were associated with greater risk of ADS and SM. In girls, school grades were only associated with SM, but not with ADS. The risk of a SM-related diagnosis declined by increasing number of best friends in the total sample and in boys.

**Table 1:** Frequencies and percentages of OHC and proposed mediators/moderators of the OHC-outcome association, by gender and outcome status.

		Total sample (n = 11,011)						Boys (n = 5,436)						Girls (n = 5,575)					
		Anxiety, depression, and self-harm			Substance misuse			Anxiety, depression, and self-harm			Substance misuse			Anxiety, depression, and self-harm			Substance misuse		
		Yes (n = 560)			Yes (n = 609)			Yes (n = 275)			Yes (n = 402)			Yes (n = 285)			Yes (n = 207)		
		n	%	*p*	n	%	*p*	n	%	*p*	n	%	*p*	n	%	*p*	n	%	*p*
OHC	No	500	4.8	*	520	5.0	*	247	4.8	*	346	6.7	*	253	4.8	*	174	3.3	*
	Yes	60	9.5		89	14.1		28	9.1		56	18.2		32	9.9		33	10.2	
Grades	1^st^ quartile	172	7.0	*	247	10.1	*	109	7.4	*	177	12.0	*	63	6.5		70	7.2	*
	2^nd^ quartile	141	5.0		157	5.6		72	5.0		105	7.3		69	5.0		52	3.8	
	3^rd^ quartile	124	4.2		123	4.2		53	4.0		79	5.9		71	4.4		44	2.7	
	4^th^ quartile	123	4.4		82	2.9		41	3.4		41	3.4		82	5.0		41	2.5	
Delinquency	No	441	4.5	*	385	4.0	*	176	4.1	*	206	4.8	*	265	4.9	*	179	3.3	*
	Yes	119	9.1		224	17.1		99	8.6		196	17.0		20	12.4		28	17.4	
Early parenthood	No	461	4.8	*	501	5.2	*	249	4.9	*	360	7.0	*	212	4.7	*	141	3.1	*
	Yes	99	7.2		108	7.8		26	8.2		42	13.2		73	6.9		66	6.2	
Number of best friends	0–1	164	5.9		197	7.1	*	80	5.5		136	9.4	*	84	6.2		61	4.5	
	2–3	248	5.0		260	5.3		120	5.2		163	7.1		128	4.9		97	3.7	
	4 or more	148	4.5		152	4.6		75	4.4		103	6.0		73	4.6		49	3.1	
Leisure time activities	None/No membership	256	5.5		251	5.4		122	5.8		157	7.4		134	5.3		94	3.7	
	Max. twice per month	77	5.5		87	6.2		35	4.6		60	7.9		42	6.5		27	4.2	
	At least once per week	227	4.6		271	5.5		118	4.6		185	7.2		109	4.5		86	3.6	

*p-value < 0.05

Logistic regression estimates of the associations between the outcomes and mediators and moderators (see S2 Fig and S5a and S5b Tables in S1 File) revealed similar patterns as in Table 1. Additionally, having four or more best friends, and structured leisure time activity at least once per week, were associated with reduced likelihood of ADS in the total sample, but not in the gender-stratified analyses.

### Potential accumulation of risk—mediation analyses

The overall and gender-stratified mediation results using the KHB method are presented in Table 2. In the full sample, the KHB analyses showed significant indirect associations between OHC and both outcomes through school grades, delinquency, and early parenthood. When stratifying by gender, the association between OHC and ADS in girls did not operate via school grades, while it did so in boys. Conversely, in boys, early parenthood was only a mediator of the association between OHC and SM, but not between OHC and ADS. For ADS, the included mediators explained 36% of the indirect association with OHC in boys, but only 15.8% in girls. For SM, the absolute difference of mediation percentages achieved by the full model was smaller, but still substantial (37% in boys and 25.6% in girls).

**Table 2:** Overall and gender-stratified mediation results.

*Outcome*: *Anxiety*, *depression*, *and self-harm*												
Exposure: OHC	Overall (n = 11,011)				Boys (n = 5,436)				Girls (n = 5,575)			
Mediating variables:	Total	Direct	Indirect	MP	Total	Direct	Indirect	MP	Total	Direct	Indirect	MP
Grades	2.08*	1.95*	1.07*	8.6	1.99*	1.82*	1.10*	13.4	2.18*	2.11*	1.03	3.9
Delinquency	2.05*	1.84*	1.11*	15.0	1.96*	1.62*	1.21*	28.4	2.14*	2.02*	1.06*	7.8
Early parenthood	2.08*	1.98*	1.05*	6.3	1.98*	1.93*	1.02	3.5	2.17*	2.04*	1.07*	8.5
Full model	2.04*	1.71*	1.19*	24.7	1.96*	1.54*	1.28*	36.0	2.12*	1.88*	1.13*	15.8
***Outcome*: *Substance misuse***												
Exposure: OHC	Overall (n = 11,011)				Boys (n = 5,436)				Girls (n = 5,575)			
Mediating variables:	Total	Direct	Indirect	MP	Total	Direct	Indirect	%	Total	Direct	Indirect	MP
Grades	3.19*	2.69*	1.19*	14.8	3.19*	2.74*	1.16*	12.8	3.32*	2.85*	1.17*	12.9
Delinquency	3.01*	2.36*	1.27*	22.0	3.09*	2.19*	1.41*	30.4	3.15*	2.82*	1.11*	9.5
Early parenthood	3.12*	2.99*	1.04*	3.7	3.08*	2.99*	1.03*	2.6	3.31*	2.94*	1.13*	9.9
Full model	3.06*	2.12*	1.45*	33.0	3.14*	2.06*	1.53*	37.0	3.07*	2.30*	1.33*	25.6

We used the KHB method, reporting Odds Ratios (ORs) for total, direct and indirect associations as well as the Mediation Percentage (MP).

*p-value < 0.05

### Potential buffering factors—moderation analyses

**Fig 1:**
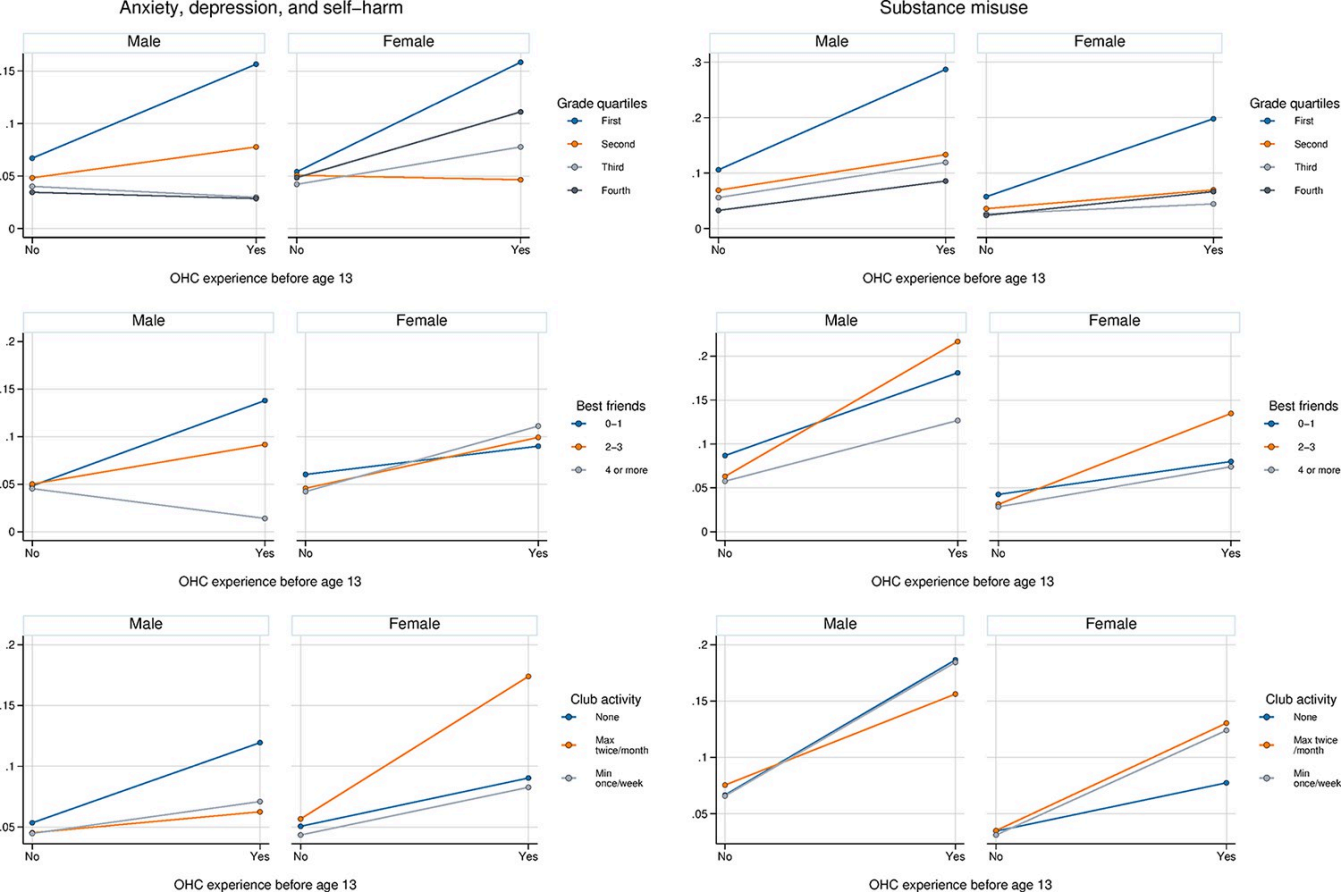
Predicted probabilities of anxiety, depression, and self-harm, and substance misuse. Shown across levels of each moderator, by gender and OHC experience.

In the two-way interaction analyses (S6a-S8b Tables in S1 File), only number of best friends was a statistically significant moderator, although not in the same direction for both outcomes (Table 3, and S7a and S7b Tables in S1 File). More specifically, while having more friends was particularly beneficial for boys with OHC experience in terms of preventing ADS, it was an exacerbating factor for SM in the total sample (S7a and S7b Tables in S1 File). The results further show that there was a tendency for higher grades to even out the risk introduced by OHC experience for both outcomes (Fig 1).

**Table 3:** P-values from likelihood ratio tests.

	Anxiety, depression, and self-harm			Substance misuse		
	Total sample (n = 11,011)	Boys (n = 5,436)	Girls (n = 5,575)	Total sample (n = 11,011)	Boys (n = 5,436)	Girls (n = 5,575)
OHC x Grades	0.12	0.23	0.15	0.27	0.60	0.38
OHC x Number of best friends	0.60	0.01*	0.49	0.04*	0.22	0.14
OHC x Leisure time activities	0.61	0.61	0.47	0.60	0.80	0.32
OHC x Grades x Gender	0.13	-	-	0.63	-	-
OHC x Number of best friends x Gender	0.12	-	-	0.39	-	-
OHC x Leisure time activities x Gender	0.64	-	-	0.90	-	-

Compared are models with interaction terms to models without interaction terms. Full results S5-S7 Tables in the S1 File.

*p-value < 0.05

Gender did not interact with OHC in relation to either outcome (ADS: interaction term p = 0.74; SM: interaction term p = 0.76). None of the three-way interaction terms (OHC x gender x moderator) were significant (Table 3).

## Discussion

The present study analysed the role of extra-familial risk and resource factors in relation to the association between childhood adversity, as indicated by OHC, and psychiatric disorders in adulthood, with a special interest in gender patterns. Such analyses provide opportunities for a deeper understanding of how vulnerability unfolds after OHC, and for identifying resource factors that may foster resilience.

The results show that OHC is prospectively associated with psychiatric disorders in adulthood. Risk appears to accumulate along pathways from OHC to adult psychiatric disorders via low school grades, delinquency, and early parenthood for both genders. The exception were low school grades in girls and early parenthood in boys, both in relation to ADS. Education appeared to be a more important mediator in boys than in girls, similar to findings from previous studies in the context of childhood socioeconomic disadvantage and depression [9, 39]. Thus, the transmission of risk along the educational pathway seems to be stronger for men. Correspondingly, educational success also bears greater protective potential for men. Why this might be the case is discussed in more detail in the context of moderation analysis later on. With regards to SM, school grades mediated a similar proportion of the association across genders, suggesting that gender patterns might be outcome-dependent.

To the best of our knowledge, our study is the first to consider delinquency and early parenthood as possible gendered pathways between OHC and psychiatric disorders. We found clear evidence of this being the case, as delinquency mediated a substantial portion of all investigated associations in boys, but not to the same extent in girls. This is somewhat different to a comparable study in which no marked gender difference in mediation via delinquent behaviour was identified in the context of adverse childhood experiences and non-medical opioid use [40]. Although not directly comparable, due to differences in the study context and outcomes, the divergence in our and Quinn et al.’s findings highlight the importance of replication and context-sensitivity in research on risk accumulation after childhood adversity. Our own findings fit with the general observation that men tend to exhibit externalising problems more frequently than women [e.g., 41], which is in line with gendered expectations around coping behaviours [42]. Conversely, early parenthood was a more important mediator in girls. Again, while we are not aware of other studies investigating exactly the same links, those exploring the most similar exposure-outcome combinations to our study, also identified gender disparities in the influence of early parenthood. For example, one study found a significant indirect effect of childhood socioeconomic disadvantage via early parenthood on heart problems in women, but not in men [14]. Taken together, the evidence suggests that early parenthood is a greater source of risk accumulation in women with experience of childhood adversity compared to men. We argue this may be due to gendered norms around care-taking as well as greater stigma and isolation related to young motherhood when compared to young fatherhood. We argue that the emergence of delinquency and early parenthood as gendered risk accumulation pathways calls for a gender-sensitive engagement with youth at risk of experiencing continued vulnerability. Examples of this could be discouragement of criminal behaviour as a demonstration of masculinity and promoting of shared responsibility in relation to early parenthood.

Overall, a greater portion of the total associations in boys could be explained through the included mediators, implying that for girls, there might be other, more important risk factors after OHC that we could not capture with our data. Drawing on previous empirical research as well as theory on gendered vulnerability processes, such factors may encompass psychosocial aspects, such as coping strategies or feelings of loneliness [40, 43, 44]. In the present study, the only available possible indicator of coping was delinquency, which, as previously noted, is more common, and socially accepted, in boys/men than in girls/women. Overall, the findings on risk accumulation suggest that assisting young individuals in avoiding unplanned parenthood (especially among young women) and delinquent behaviour (especially among young men) represents a gender-sensitive approach to disrupting some of the mechanisms through which childhood adversity can lead to adult psychiatric disorders.

We were not able to clearly identify resource factors that buffer the impact of OHC on psychiatric disorders. Only one of the two-way-interaction terms was significant, namely that between OHC and friendship, but the direction was inconsistent: The results indicate that increasing number of friends among boys evens out the risk of ADS, bringing those with OHC placement on a similar risk level to those without. In the total sample, the reverse was true: a greater number of friends disproportionately increased the risk of SM for those with OHC experience. This points to the importance of context–not only the number, but also the quality of friendships matters for increases or decreases in the risk of negative outcomes [45]. In contrast to existing evidence on the importance of social support for girls and women [e.g., 25, 26], no moderation was observed in girls. One possible explanation is a shift in the positive influence of social connection throughout the life course. During adolescence, gender differences in friendship values intensify, and while girls value psychological provisions such as emotional support more than boys [46], they may also experience more stress around nurturing their relationships [47]. It is possible that for those with a large number of friendships, the pressure to preserve these relationships neutralises their positive potential in girls, while boys tend to face lower expectations around prosocial behaviour in the upkeep of social relationships [48].

Despite the lack of statistical significance, our results indicated that higher school grades may even out the risk of psychiatric disorders. High school grades can be considered an early proxy of positive educational achievement, with good predictive power for career trajectories later on in life [49]. While other studies have repeatedly shown the general value of education as a resource and its role as mediator, studies do not necessarily find it to be a buffer in the context of childhood adversity [50–53]. Our findings also support the view that education is more important in its function as mediating pathway, but still suggest it can also act as buffer, i.e., in this context as a resource that is particularly powerful following childhood adversity. Since it is well established that adverse childhood experiences, even before a clinical problem is manifest, may translate into behavioural problems interfering with school performance [54], it seems reasonable to assume that education is primarily a mediator. However, in cases where a child is enabled to “beat the odds” and still do well in school, despite heightened difficulties in the home environment, they may draw even more advantage from educational success than a child without such experiences. While the present study failed to identify any significant gender differences in moderation, the results suggest that success in school was especially helpful in preventing negative outcomes in boys, which could be due to gendered expectations around schooling. Girls typically experience more stress in relation to school performance [55]. This could constitute a counterweight to the benefit of greater access to resources provided by higher education, at least in relation to ADS. Alternatively, even though women in this cohort entered the workforce in larger numbers than previous generations, school grades may still have been more directly linked to future SES in boys. This is may be due to, for example, more limited career opportunities for women, with persisting horizontal and vertical labour market segregation by gender [56].

Lastly, in contrast to previous research [27], leisure time activities in adolescence did not act as a buffer in the association between OHC and adult psychiatric disorders in our data, apart from possibly in boys in relation to ADS. This lack of a clear association may be due to low statistical power, and that leisure time activities were limited to organised club memberships. Future studies should consider both structured and unstructured forms of leisure time activity.

### Strengths and limitations

This study has the benefit of an unusual combination of information: verified accounts of childhood adversity, high-quality register data with a long-follow up for psychiatric disorders, and information on a range of extra-familial factors that may act as risks or resources in this context. To the best of our knowledge, our study is the first to explore the combination of these factors from a gender perspective, thereby providing a valuable addition to the research field.

Although this study applied causal inference methods (i.e., mediation analysis), we cannot claim causality. For example, part of the OHC placements extended beyond age 13, meaning that the temporal order of the exposure and mediators and moderators cannot be ascertained. Similarly, many mental health problems can have their onset in early adolescence or even childhood, especially conditions related to anxiety [57]. This means that reciprocal causality is a likely part of the picture. This is a general problem with observational studies, and renders replication of findings in different setting particularly important. The identified dose-response relationships however indicate causal relationships, supported by the fact that our findings are also largely aligned with previous research. Furthermore, by using inpatient data, we only capture the most severe cases, and rely on healthcare use as a proxy for healthcare need. A more comprehensive picture might emerge if we would consider milder cases of mental health problems. While we have access to data on outpatient care, this register is only available from 2001 (age 48) onward. Considering that the onset of mental ill-health typically occurs earlier in life, the contribution of adding outpatient data is likely to be minimal.

Another limitation is the crude information on the exposure, as well as on risk and resource factors. For example, data do not allow us to discern the exact cause of OHC. Furthermore, we did not consider length or timing of stay, in part due to lack of information, and in part in order to no decrease group sizes and lose statistical power. Moreover, we are not able to identify what part of the observed associations is due to the underlying reasons for placement, and what is due to the experience of OHC itself. We argue that this is not a major problem, since different forms of early adversity often cluster together [58], rendering a general proxy for severe adversity a good match for the purpose of our study. Having access to a verified proxy of adversity was more important than understanding its exact nature, and allowed us to focus on gender patterns in risk accumulation and resource factors in this context. However, given that our findings suggest that the processes under investigation might be partly outcome- and gender-specific, it may also be the case that they are exposure-specific. Consequently, a more detailed engagement with the exposure would provide additional insights, making it a relevant starting point for future studies with access to such information. Furthermore, attrition and missing information in this sample are not random. For example, missing information on one of the mediators or moderators was more common among cohort members with OHC placement before age 13 (S1 Table in S1 File). Consequently, the sample became more selected, which likely implies a potential underestimation of the observed associations.

Lastly, we would like to address the generalisability of our findings. OHC placements in Sweden during the 50s and 60s were different from OHC placements today, with the child welfare system shifting from a more interventionist stance to a focus on family reunification, and a more participatory approach to deciding child welfare measures [59, 60]. Accordingly, the prevalence of OHC was higher than it is today, which implies a more heterogeneous group, for example in terms of case severity. Based on this, we assume that if conducting the same study in a younger cohort, we would find even stronger associations. Lastly, it is worth noting the possibility of gender bias in our outcome, with, e.g., some types of psychiatric disorders such as depression remaining underdiagnosed especially in men [61], which may have led to an underestimate of the observed associations. We have tried to limit this problem by including both outcomes that are typically more frequently observed in women, and outcomes that are typically more frequently observed in men.

## Conclusions

This study shows how extra-familial risk factors accumulate along pathways linking OHC to adult psychiatric disorders. This occurs in an outcome- and gender-dependent fashion, with early parenthood being a particularly important risk factor in girls with OHC experience, and delinquency a particularly important risk factor in boys. We found limited evidence for friendships and higher school grades acting as extra-familial resource factors that even out the excess risk of psychiatric disorders among those exposed to OHC. In conclusion, there is much to suggest that vulnerability and resilience can be understood as context-dependent and gendered processes, the details of which warrant further investigation.

## Supporting information

S1 File Flow chart, variable descriptions, and regression results. (PDF)
