# Supplementary material for: The role of extra-familial factors in adolescence for the association between out-of-home care and adult psychiatric disorders–A birth cohort study
Source: PLoS One. 2025 Jan 28;20(1):e0318231. doi: 10.1371/journal.pone.0318231 (PMC11774388; doi:10.1371/journal.pone.0318231)
Supplement: S1 File — (PDF) [file pone.0318231.s001.pdf]

Supporting information

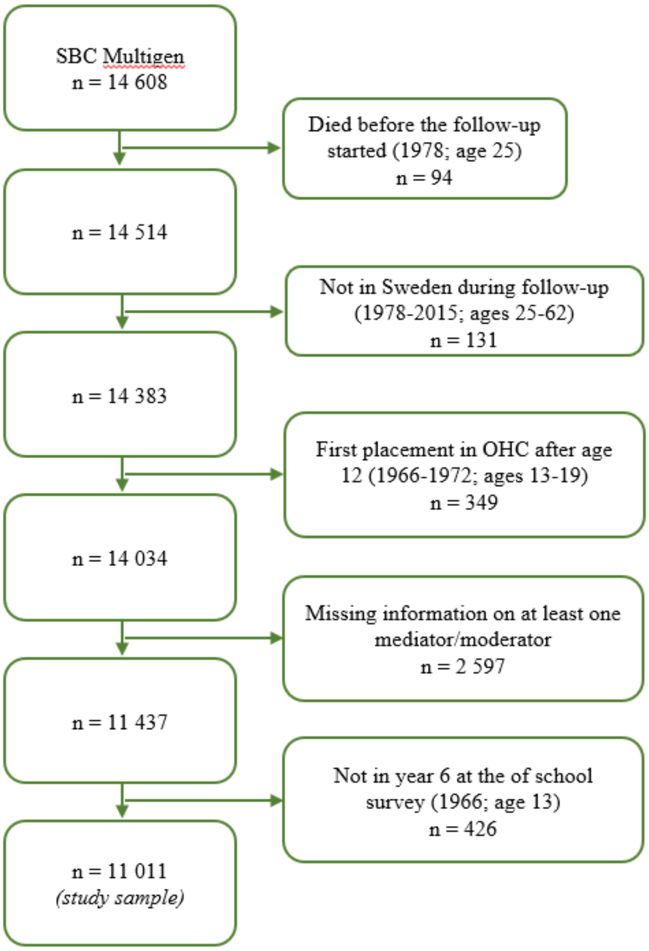

S1 Fig. Flow-chart of the study sample

S1 Table. Frequencies of OHC placement before age 13 by reasons for exclusion from study sample.

	Included	Excluded	
OHC placement before age 13		Migrated or died before 1978	Missing information
No	10,382 (90.90%)	146 (1.27%)	893 (7.82%)
Yes	629 (88.72%)	6 (0.84%)	74 (10.44%)
Total	11,011 (90.77%)	152 (1.26%)	967 (7.97)

S2 Table. Overview of diagnostic codes (ICD-10) included in the outcomes used in this study.

**ICD-10 Category**	**ICD-10 codes**	**ICD-10 subcategory**	**ICD-10 code**
**Outcome 1: Anxiety, depression, and self-harm**			
Mood (affective) disorders (F30-F39)			
Depressive episode	F32		
Recurrent depressive disorder	F33		
Neurotic, stress-related and somatoform disorders (F40-F48)			
Phobic anxiety disorders	F40	Agoraphobia	F400
		Social phobias	F401
		Specific phobias	F402
Other anxiety disorders	F41	Panic disorder	F410
		Generalised anxiety disorder	F411
Obsessive-compulsive disorder	F42	Predominantly obsessional thoughts or ruminations	F420
		Predominantly compulsive acts	F421
Reaction to severe stress, and adjustment disorders	F43	Acute stress reaction	F430
		Post-traumatic stress disorder	F431
External causes of morbidity and mortality			
Intentional self-harm	X60-X84		
Event of undetermined intent	Y10-Y34		
**Outcome 2: Substance misuse**			
Disorders of other endocrine glands (E20-E35)			
		Alcohol-induced pseudo-cushing syndrome	E244
Mental and behavioural disorders due to psychoactive substance use (F10-F19)			
Mental and behavioural disorders due to use of alcohol	F10		
Mental and behavioural disorders due to use of opioids	F11		
Mental and behavioural disorders due to use of cannabinoids	F12		
Mental and behavioural disorders due to use of sedatives or hypnotics	F13		
Mental and behavioural disorders due to use of cocaine	F14		
Mental and behavioural disorders due to use of other stimulants, including caffeine	F15		
Mental and behavioural disorders due to use of hallucinogens	F16		
Mental and behavioural disorders due to use of volatile solvents	F18		
Mental and behavioural disorders due to multiple drug use and use of other psychoactive substances	F19		
Other degenerative diseases of the nervous system (G30-G32)			
		Degeneration of nervous system due to alcohol	G312
Polyneuropathies and other disorders of the peripheral nervous system (G60-G64)			
		Alcoholic polyneuropathy	G621

S2 Table continued. Overview of diagnostic codes (ICD 10) included in the outcomes used in this study

Diseases of myoneural junction and muscle (G70-G73)			
		Alcoholic myopathy	G721
Other forms of heart disease (I30-I52)			
		Alcoholic cardiomyopathy	I426
Diseases of oesophagus, stomach and duodenum (K20-K31)			
		Alcoholic gastritis	K292
Diseases of liver (K70-K77)			
Alcoholic liver disease	K70		
Disorders of gallbladder, biliary tract and pancreas (K80-K87)			
		Alcohol-induced acute pancreatitis	K852
		Drug-induced acute pancreatitis	K860
Maternal care related to the fetus and amniotic cavity and possible delivery problems (O30-O48)			
		Maternal care for (suspected) damage to fetus from alcohol	O354
		Maternal care for (suspected) damage to fetus by drugs	O355
Poisoning by drugs, medicaments and biological substances (T36-T50)			
Poisoning by narcotics and psychodysleptics [hallucinogens]	T40		
		Poisoning by barbiturates	T423
		Poisoning by benzodiazepines	T424
		Poisoning by other antiepileptic and sedative-hypnotic drugs	T426
		poisoning by Psychostimulants with abuse potential	T436
Toxic effects of substances chiefly nonmedicinal as to source (T51-T65)			
Toxic effect of alcohol	T51		
Accidental poisoning by and exposure to noxious substances (X40-X49)			
Accidental poisoning by and exposure to alcohol	X45		
Intentional self-harm (X60-X84)			
Intentional self-poisoning by and exposure to other and unspecified drugs, medicaments and biological substances	X64		
Event of undetermined intent (Y10-Y34)			
Poisoning by and exposure to alcohol, undetermined intent	Y15		
Supplementary factors related to causes of morbidity and mortality classified elsewhere (Y90-Y98)			
Evidence of alcohol involvement determined by blood alcohol level	Y90		
Evidence of alcohol involvement determined by level of intoxication	Y91		
Persons encountering health services for specific procedures and health care (Z40-Z54)			
		Alcohol rehabilitation	Z502
		Drug rehabilitation	Z503
Persons encountering health services in other circumstances (Z70-Z76)			
		Alcohol abuse counselling and surveillance	Z714
		Drug abuse counselling and surveillance	Z715
		Problems related to alcohol use	Z721
		Problems related to drug use	Z722

S3 Table. Overview of mediators and moderators

Variable	Description	Operationalisation	Data Source
Grades	The cohort members' average grades in the spring term of year 6.	Categorical mediator and moderator (Grade quartiles, with the first quartile being the lowest and the fourth the highest).	Marks Register
Delinquency	Refers to ages 14 to 21. The variable was coded as 0 for those who never committed a crime (not included: narcotics related offences), and 1 for those who committed a least one crime that was not narcotics related.	Binary mediator (No/Yes)	National Crime Register
Early parenthood	This variable was coded 1 for becoming a parent for the first time between age 14 and 21, and 0 otherwise.	Binary mediator (No/Yes)	Total Population Register
Number of best friends	Cohort members were asked to vote for their three best friends in class. Based on the number of votes received.	Categorical moderator (0-1; 2-3; 4 or more)	The School Survey (1966)
Leisure time activities	This variable captures the cohort members' engagement in clubs or associations.	Categorical moderator (None/No memberships; Max. twice/month; At least once/week)	The School Survey (1966)

S4a Table. HRs and 95% CIs from Cox regression of anxiety, depression, and self-harm on different predictors (bivariate associations).

		**Overall (n=10,827)**	**Boys (n=5,359)**	**Girls (n=5,468)**
OHC	Yes	1.89 (1.40, 2.54)*	1.70 (1.09, 2.66)*	2.06 (1.38, 3.08)*
Grades	1st quartile	(reference category)	(reference category)	(reference category)
	2nd quartile	0.75 (0.60, 0.96)*	0.71 (0.52, 0.97)*	0.83 (0.58, 1.20)
	3rd quartile	0.60 (0.47, 0.77)*	0.56 (0.39, 0.79)*	0.67 (0.47, 0.97)*
	4th quartile	0.61 (0.47, 0.78)*	0.44 (0.30, 0.65)*	0.78 (0.55, 1.12)
Delinquency		2.07 (1.67, 2.57)*	2.17 (1.67, 2.82)*	2.74 (1.68, 4.49)*
Early parenthood		1.52 (1.21, 1.92)*	1.82 (1.19, 2.77)*	1.45 (1.10, 1.93)*
Number of best friends	0-1	(reference category)	(reference category)	(reference category)
	2-3	0.85 (0.69, 1.04)	0.89 (0.66, 1.21)	0.80 (0.60, 1.08)
	4 or more	0.75 (0.59, 0.95)*	0.76 (0.55, 1.07)	0.73 (0.52, 1.02)
Leisure time activities	None/No membership	(reference category)	(reference category)	(reference category)
	Max twice/month	0.96 (0.72, 1.27)	0.85 (0.57, 1.26)	1.09 (0.73, 1.62)
	At least once/week	0.88 (0.73, 1.07)	0.82 (0.63, 1.08)	0.94 (0.72, 1.23)

*p-value < 0.05

S4b Table. HRs and 95% CIs from Cox regression of substance misuse on different predictors (bivariate associations).

		**Overall (n=10,938)**	**Boys (n=5,403)**	**Girls (n=5,535)**
OHC	Yes	2.81 (2.21, 3.57)*	2.67 (1.97, 3.63)*	3.18 (2.15, 4.70)*
Grades	1st quartile	(reference category)	(reference category)	(reference category)
	2nd quartile	0.53 (0.43, 0.66)*	0.57 (0.44, 0.74)*	0.53 (0.36, 0.76)*
	3rd quartile	0.41 (0.33, 0.51)*	0.51 (0.39, 0.67)*	0.35 (0.24, 0.53)*
	4th quartile	0.29 (0.23, 0.38)*	0.30 (0.21, 0.42)*	0.35 (0.23, 0.52)*
Delinquency		4.38 (3.68, 5.21)*	3.61 (2.94, 4.42)*	5.03 (3.23, 7.85)*
Early parenthood		1.50 (1.21, 1.86)*	2.08 (1.51, 2.88)*	1.87 (1.38, 2.55)*
Number of best friends	0-1	(reference category)	(reference category)	(reference category)
	2-3	0.74 (0.61, 0.89)*	0.73 (0.57, 0.92)*	0.84 (0.60, 1.17)
	4 or more	0.64 (0.51, 0.79)*	0.63 (0.48, 0.82)*	0.65 (0.43, 0.96)*
Leisure time activities	None/ No membership	(reference category)	(reference category)	(reference category)
	Max twice/month	1.18 (0.92, 1.52)	1.12 (0.83, 1.53)	1.09 (0.69, 1.71)
	At least once/week	1.04 (0.87, 1.25)	1.03 (0.82, 1.28)	0.95 (0.70, 1.29)

*p-value < 0.05

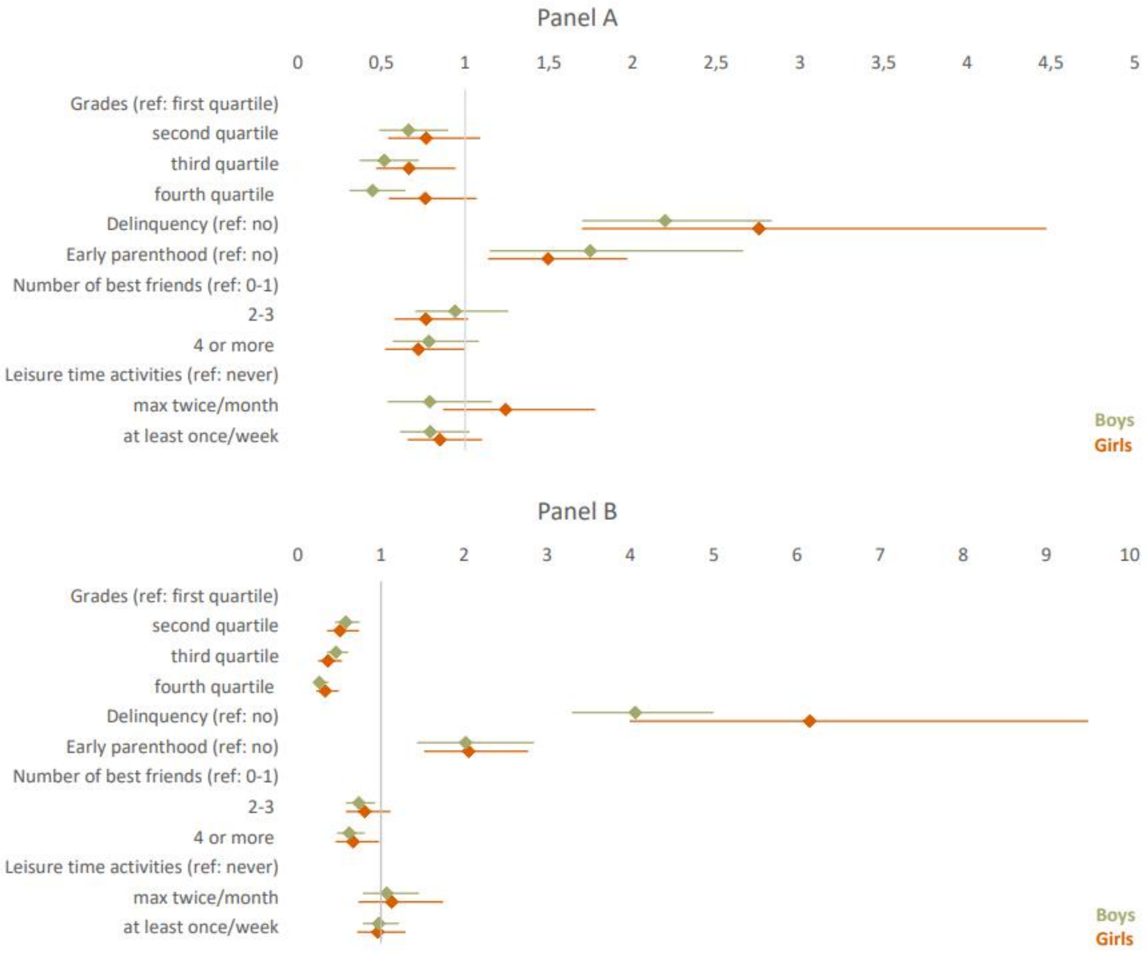

S2 Fig. OR plots of bivariate, gender-stratified associations of mediators and moderators with anxiety, depression, and self-harm (Panel A), and mediators and moderators with substance misuse (Panel B).

S5a Table. ORs and 95% CIs from logistic regression of anxiety, depression, and self-harm on different predictors (bivariate associations).

		**Overall (n=11,011)**	**Boys (n=5,436)**	**Girls (n=5,575)**
Grades	1st quartile	(reference category)	(reference category)	(reference category)
	2nd quartile	0.70 (0.56, 0.88)*	0.66 (0.49, 0.90)*	0.77 (0.54, 1.09)
	3rd quartile	0.58 (0.46, 0.74)*	0.52 (0.37, 0.72)*	0.67 (0.47, 0.94)*
	4th quartile	0.60 (0.47, 0.76)*	0.45 (0.31, 0.64)*	0.76 (0.54, 1.07)
Delinquency		2.09 (1.70, 2.58)*	2.19 (1.70, 2.83)*	2.76 (1.70, 4.47)*
Early parenthood		1.54 (1.23, 1.92)*	1.75 (1.15, 2.66)*	1.50 (1.14, 1.97)*
Number of best friends	0-1	(reference category)	(reference category)	(reference category)
	2-3	0.85 (0.69, 1.04)	0.94 (0.70, 1.26)	0.77 (0.58, 1.02)
	4 or more	0.75 (0.60, 0.94)*	0.78 (0.57, 1.08)	0.72 (0.52, 1.00)
Leisure time activities	None/ No membership	(reference category)	(reference category)	(reference category)
	Max twice/month	0.99 (0.76, 1.29)	0.79 (0.54, 1.16)	1.24 (0.87, 1.78)
	At least once/week	0.82 (0.68, 0.99)*	0.79 (0.61, 1.03)	0.85 (0.66, 1.10)

*p-value < 0.05

S5b Table. ORs and 95% CIs from logistic regression of substance misuse on different predictors (bivariate associations).

		**Overall (n=11,011)**	**Boys (n=5,436)**	**Girls (n=5,575)**
Grades	1st quartile	(reference category)	(reference category)	(reference category)
	2nd quartile	0.53 (0.43, 0.65)*	0.58 (0.45, 0.74)*	0.51 (0.35, 0.74)*
	3rd quartile	0.39 (0.31, 0.48)*	0.46 (0.35, 0.61)*	0.36 (0.25, 0.53)*
	4th quartile	0.27 (0.21, 0.34)*	0.26 (0.18, 0.37)*	0.33 (0.22, 0.49)*
Delinquency		4.98 (4.17, 5.93)*	4.06 (3.30, 5.00)*	6.16 (3.99, 9.50)*
Early parenthood		1.55 (1.25, 1.92)*	2.02 (1.43, 2.84)*	2.05 (1.52, 2.77)*
Number of best friends	0-1	(reference category)	(reference category)	(reference category)
	2-3	0.73 (0.60, 0.89)*	0.73 (0.58, 0.93)*	0.80 (0.58, 1.12)
	4 or more	0.64 (0.51, 0.79)*	0.62 (0.47, 0.81)*	0.67 (0.46, 0.98)*
Leisure time activities	None/ No membership	(reference category)	(reference category)	(reference category)
	Max twice/month	1.15 (0.90, 1.48)	1.07 (0.78, 1.46)	1.13 (0.73, 1.75)
	At least once/week	1.01 (0.85, 1.21)	0.97 (0.78, 1.22)	0.96 (0.71, 1.30)

*p-value < 0.05

S6a Table. Logistic regression of anxiety, depression, and self-harm on OHC, with interaction terms between OHC and grades (ORs and 95% CIs presented for the overall sample and stratified by gender).

		**Overall (n=11,011)**		**Boys (n=5,436)**		**Girls (n=5,575)**	
		Model 1	Model 2	Model 1	Model 2	Model 1	Model 2
OHC		1.95 (1.47, 2.59)*	2.83 (1.89, 4.24)*	1.82 (1.20, 2.75)*	2.58 (1.50, 4.46)*	2.11 (1.43, 3.12)*	3.29 (1.79, 6.06)*
Grades	2nd quartile	0.71 (0.57, 0.90)*	0.79 (0.62, 1.01)	0.67 (0.49, 0.91)*	0.71 (0.51, 0.98)*	0.80 (0.56, 1.14)	0.94 (0.64, 1.38)
	3rd quartile	0.60 (0.47, 0.76)*	0.65 (0.51, 0.84)*	0.53 (0.38, 0.74)*	0.58 (0.41, 0.83)*	0.70 (0.49, 0.99)*	0.77 (0.52, 1.13)
	4th quartile	0.63 (0.50, 0.80)*	0.67 (0.52, 0.87)*	0.46 (0.32, 0.67)*	0.50 (0.34, 0.73)*	0.82 (0.58, 1.16)	0.89 (0.61, 1.29)
OHC# Grades	Yes#2nd quartile		0.45 (0.21, 0.96)*		0.64 (0.24, 1.71)		0.28 (0.08, 0.92)*
	Yes#3rd quartile		0.50 (0.22, 1.12)		0.28 (0.06, 1.32)		0.58 (0.21, 1.61)
	Yes#4th quartile		0.64 (0.25, 1.65)		0.32 (0.04, 2.56)		0.74 (0.24, 2.32)

*p-value < 0.05; The LR test comparing Model 1 and Model 2 was non-significant in the overall sample (p-value 0.12), in boys (p-value 0.23), and in girls (p-value 0.15).

S6b Table. Logistic regression of substance misuse on OHC, with interaction terms between OHC and grades (ORs and 95% CIs presented for the overall sample and stratified by gender)

		Overall (n=11,011)		Boys (n=5,436)		Girls (n=5,575)	
		Model 1	Model 2	Model 1	Model 2	Model 1	Model 2
OHC		2.69 (2.11, 3.44)*	3.41 (2.42, 4.80)*	2.75 (2.01, 3.76)*	3.39 (2.19, 5.26)*	2.85 (1.91, 4.24)*	4.04 (2.30, 7.13)*
Grades	2nd quartile	0.54 (0.44, 0.67)*	0.59 (0.47, 0.73)*	0.59 (0.46, 0.76)*	0.63 (0.48, 0.82)*	0.54 (0.37, 0.78)*	0.61 (0.41, 0.92)*
	3rd quartile	0.40 (0.32, 0.51)*	0.43 (0.34, 0.55)*	0.48 (0.36, 0.63)*	0.50 (0.37, 0.67)*	0.39 (0.26, 0.57)*	0.44 (0.29, 0.68)*
	4th quartile	0.29 (0.22, 0.37)*	0.30 (0.23, 0.39)*	0.28 (0.20, 0.39)*	0.29 (0.20, 0.41)*	0.37 (0.25, 0.55)*	0.40 (0.26, 0.62)*
OHC# Grades	Yes#2nd quartile		0.60 (0.32, 1.11)		0.61 (0.28, 1.33)		0.50 (0.17, 1.42)
	Yes#3rd quartile		0.59 (0.29, 1.19)		0.67 (0.28, 1.65)		0.42 (0.13, 1.40)
	Yes#4th quartile		0.84 (0.33, 2.11)		0.81 (0.22, 3.00)		0.72 (0.19, 2.75)

*p-value < 0.05; The LR test comparing Model 1 and Model 2 was non-significant in the overall sample (p-value 0.27), in boys (p-value 0.60), and in girls (p-value 0.38).

S7a Table. Logistic regression of anxiety, depression, and self-harm on OHC, with interaction terms between OHC and number of best friends (ORs and 95% CIs presented for the overall sample and stratified by gender).

		**Overall (n=11,011)**		**Boys (n=5,436)**		**Girls (n=5,575)**	
		Model 1	Model 2	Model 1	Model 2	Model 1	Model 2
OHC		2.04 (1.54, 2.71)*	2.29 (1.46, 3.59)*	1.95 (1.29, 2.93)*	3.15 (1.76, 5.66)*	2.14 (1.45, 3.15)*	1.54 (0.75, 3.18)
Number of best friends	0-1	(ref)	(ref)	(ref)	(ref)	(ref)	(ref)
	2-3	0.87 (0.71, 1.06)	0.88 (0.71, 1.09)	0.96 (0.72, 1.29)	1.04 (0.76, 1.43)	0.78 (0.59, 1.04)	0.75 (0.55, 1.01)
	4 or more	0.77 (0.62, 0.97)*	0.80 (0.63, 1.02)	0.81 (0.59, 1.12)	0.94 (0.66, 1.32)	0.74 (0.53, 1.02)	0.69 (0.49, 0.97)*
OHC#2-3			0.92 (0.49, 1.72)		0.61 (0.25, 1.45)		1.49 (0.59, 3.78)
OHC#4 or more			0.67 (0.30, 1.49)		0.10 (0.01, 0.76)*		1.83 (0.65, 5.15)

*p-value < 0.05; The LR test comparing Model 1 and Model 2 was non-significant in the overall sample (p-value 0.60), and in girls (p-value 0.49), but significant in boys (p-value 0.01).

S7b Table. Logistic regression of substance misuse on OHC, with interaction terms between OHC and number of best friends (ORs and 95% CIs presented for the overall sample and stratified by gender).

		**Overall (n=11,011)**		**Boys (n=5,436)**		**Girls (n=5,575)**	
		Model 1	Model 2	Model 1	Model 2	Model 1	Model 2
OHC		3.02 (2.37, 3.85)*	2.22 (1.46, 3.38)*	2.95 (2.16, 4.03)*	2.32 (1.40, 3.87)*	3.27 (2.21, 4.84)*	1.96 (0.90, 4.24)
Number of best friends	0-1	(ref)	(ref)	(ref)	(ref)	(ref)	(ref)
	2-3	0.76 (0.63, 0.92)*	0.69 (0.56, 0.85)*	0.77 (0.60, 0.97)*	0.71 (0.55, 0.92)*	0.83 (0.60, 1.16)	0.73 (0.51, 1.04)
	4 or more	0.67 (0.54, 0.83)*	0.65 (0.52, 0.82)*	0.65 (0.50, 0.86)*	0.64 (0.48, 0.85)*	0.70 (0.47, 1.02)	0.66 (0.44, 0.99)
OHC#2-3			1.95 (1.13, 3.36)*		1.77 (0.88, 3.53)		2.47 (0.96, 6.31)
OHC#4 or more			1.08 (0.54, 2.18)		1.02 (0.42, 2.49)		1.40 (0.43, 4.53)

*p-value < 0.05; The LR test comparing Model 1 and Model 2 was significant in the overall sample (p-value 0.04), but non-significant in boys (p-value 0.22), and in girls (p-value 0.14).

S8a Table. Logistic regression of anxiety, depression, and self-harm on OHC, with interaction terms between OHC and leisure time activities (ORs and 95% CIs presented for the overall sample and stratified by gender).

		**Overall (n=11,011)**		**Boys (n=5,436)**		**Girls (n=5,575)**	
		Model 1	Model 2	Model 1	Model 2	Model 1	Model 2
OHC		2.07 (1.56, 2.74)*	2.12 (1.42, 3.16)*	1.96 (1.30, 2.96)*	2.40 (1.38, 4.20)*	2.15 (1.50, 3.16)*	1.86 (1.04, 3.22)*
Leisure time activities	None/No Membership	(ref)	(ref)	(ref)	(ref)	(ref)	(ref)
	Max twice/month	1.00 (0.77, 1.30)	0.97 (0.73, 1.28)	0.80 (0.55, 1.18)	0.84 (0.56, 1.25)	1.23 (0.86, 1.76)	1.13 (0.76, 1.67)
	At least once/week	0.83 (0.69, 1.00)	0.84 (0.69, 1.02)	0.80 (0.61, 1.03)	0.83 (0.63, 1.09)	0.86 (0.66, 1.11)	0.85 (0.65, 1.12)
OHC	#Max twice/month		1.31 (0.58, 2.95)		0.59 (0.12, 2.83)		1.88 (0.68, 5.20)
	#At least once/week		0.85 (0.45, 1.58)		0.68 (0.28, 1.63)		1.06 (0.44, 2.60)

*p-value < 0.05; The LR test comparing Model 1 and Model 2 was non-significant in the overall sample (p-value 0.61), in boys (p-value 0.61), and in girls (p-value 0.47)

S8b Table. Logistic regression of substance misuse on OHC, with interaction terms between OHC and leisure time activities (ORs and 95% CIs presented for the overall sample and stratified by gender).

		**Overall (n=11,011)**		**Boys (n=5,436)**		**Girls (n=5,575)**	
		Model 1	Model 2	Model 1	Model 2	Model 1	Model 2
OHC		3.13 (2.46, 3.99)*	2.84 (1.96, 4.12)*	3.10 (2.27, 4.22)*	3.22 (2.01, 5.14)*	3.32 (2.25, 4.91)*	2.34 (1.25, 4.40)*
Leisure time activities	None/No membership	(ref)	(ref)	(ref)	(ref)	(ref)	(ref)
	Max twice /month	1.17 (0.91, 1.51)	1.17 (0.90, 1.54)	1.11 (0.81, 1.51)	1.14 (0.83, 1.59)	1.11 (0.72, 1.72)	1.01 (0.62, 1.65)
	At least once/week	1.03 (0.86, 1.23)	1.00 (0.82, 1.21)	0.99 (0.79, 1.23)	0.99 (0.78, 1.26)	0.98 (0.73, 1.32)	0.90 (0.65, 1.24)
OHC	# Max twice /month		0.95 (0.44, 2.06)		0.71 (0.24, 2.11)		1.76 (0.56, 5.57)
	#At least once/week		1.27 (0.76, 2.12)		1.00 (0.52, 1.92)		1.88 (0.79, 4.45)

*p-value < 0.05; The LR test comparing Model 1 and Model 2 was non-significant in the overall sample (p-value 0.60), in boys (p-value 0.80), and in girls (p-value 0.32).
